# Mitogenome of the extinct helmeted musk ox, *Bootherium bombifrons*

**DOI:** 10.1080/23802359.2016.1250136

**Published:** 2016-11-11

**Authors:** Abagael Rosemary West

**Affiliations:** Division of Paleontology, American Museum of Natural History, New York, NY, USA

**Keywords:** *Bootherium*, *Ovibos*, Alaska, musk ox, Arctic

## Abstract

The complete mitochondrial genome of the extinct musk ox *Bootherium bombifrons* is presented for the first time. Phylogenetic analysis supports placement of *Bootherium* as sister to the living musk ox, *Ovibos moschatus*, in agreement with morphological taxonomy. SNPs identified in the COI-5p region provide a tool for the identification of *Bootherium* among material, which is not morphologically diagnosable, for example postcrania, coprolites, and archaeological specimens, and/or lacks precise stratigraphic control, like many from glacial alluvium and in placer mines.

The extant musk ox, *Ovibos moschatus*, represents only a relict of the Pleistocene diversity of Ovibovini. The helmeted musk ox, *Bootherium*, was endemic to North America and widespread through Canada and the continental US from mid-Pleistocene until ∼10ka, and by extension is inferred to have occupied a more varied habitat than *Ovibos*, which is now restricted to the high Nearctic.

Ancient DNA provides a wealth of data to test systematic and spatiotemporal hypotheses. In the case of *Ovibos*, ancient and modern DNA have been used to reconstruct historical phylogeography and genetic diversity across their Holarctic range (MacPhee et al. 2005; MacPhee & Greenwood 2007; Campos et al. 2010a, 2010b). Outcomes of this work indicate that *Ovibos* were once much more populous (indeed, the fossil record spreads around the entire Arctic), but also that a bottleneck around the time musk oxen became restricted to their current range (∼20ka) has left their extant population genetically depauperate.

*Bootherium* specimens AMNH-FM 142459, 145489, and 14590 were collected in the mid 20th century from placer mines near Fairbanks, Alaska (N 65.5°, W 148.5°). Target enrichment and IonTorrent sequencing (MYcroarray^®^, Ann Arbor, MI) with baits from an *Ovibos moschatus* sequence (Hassanin et al. 2009) were conducted on DNA extracted from these three specimens. After assembly in Mira 4.0 (Chevreux et al. 1999) and CAP3 (Huang & Madan 1999), BLAST + searches followed methods in Kolokotronis et al. (2016). Final assembly used Geneious 8.1.8 (Kearse et al. 2012). A complete mitogenome, 16,496 bp long, comprising 13 protein coding genes, two rRNA and 22 tRNA, and control region, was assembled from AMNH-FM 145490 and is on GenBank under accession number KX982584.

Protein-coding and tRNA genes for the three specimens were aligned against published mitogenomes from 13 caprines and two outgroups. Missing data in the other two *Bootherium* specimens is in the control region, cytochrome B, and COI. Control region reads were aligned separately from the rest of the genes. Results of the phylogenetic analysis, shown in Figure 1, support the sister-group relationship of *Bootherium* to *Ovibos*. The *Bootherium* mitogenome shows no major rearrangements. One SNP was identified as diagnosing *Bootherium* in the barcoding region COI-5p, by comparison to the rest of Caprini, as well as to other bovids including the American bison, with which *Bootherium* shared much of its range. The divergence time between *Bootherium* and *Ovibos* was estimated at ∼250 kyr BP, based on lognormal relaxed molecular clocks, and calibrations from Hassanin et al. (2012). Fossil ovibovine taxonomy centres on a long-established paradigm (Guthrie 1992 and references therein) of horn core morphology. Campos et al. (2010b) obtained the first sequences from three extinct musk ox taxa; fragments of the two genes from the *Bootherium* supported the morphological taxonomy, placing it as a sister genus to *Ovibos.* However, population dynamics studies on the scale of the *Ovibos* work are not possible with such limited data. The *Bootherium* mitogenome will contribute significantly to the available extinct ovibovid molecular information, and in future towards a deeper understanding of extinction dynamics in North America throughout the late Pleistocene.

**Figure 1.**
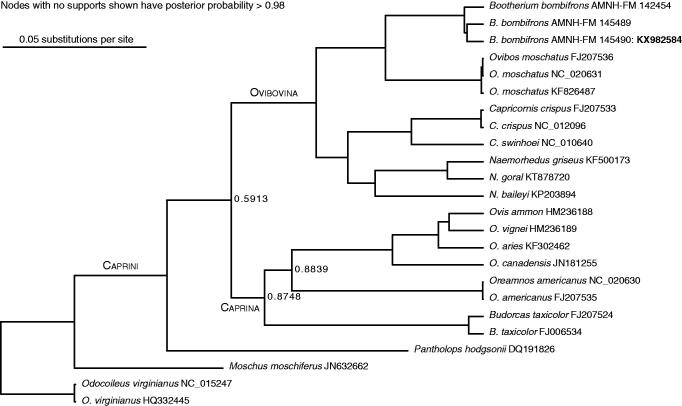
Maximum posterior probability tree of Caprini, built from complete mitogenomes excluding the control region. Multiple sequence alignment was carried out using Mauve (Darling et al. 2004) and ClustalW (Larkin et al. 2007). Trees were built in BEAST (Drummond et al. 2012) and PAUP (Swofford 1991). Model partitions follow the scheme used for *Ovis* by Sanna et al. (2015).
